# Energiekrise und Lieferstopp für Gas: Auswirkungen auf die Betriebe in Deutschland

**DOI:** 10.1007/s10273-022-3211-7

**Published:** 2022-06-24

**Authors:** Christian Kagerl, Michael Moritz, Duncan Roth, Jens Stegmaier, Ignat Stepanok, Enzo Weber

**Affiliations:** 1 Institut für Arbeitsmarkt- und Berufsforschung (IAB), Regensburger Straße 104, 90478 Nürnberg, Deutschland; 2 Regionales Forschungsnetz, Institut für Arbeitsmarkt- und Berufsforschung (IAB), Regensburger Straße 104, 90478 Nürnberg, Deutschland; 3 Forschungsbereich A1, Inst. f. Arbeitsmarkt- und Berufsforschung, Regensburger Str. 100, 90478 Nürnberg, Deutschland

**Keywords:** Q34, Q31, Q41

## Abstract

Seit 2021 steigen die Energiepreise kräftig an. Mit Beginn des russischen Kriegs gegen die Ukraine sind sie noch einmal in die Höhe geschnellt. Neben Lieferkettenstörungen und Exportrückgängen dämpft das die wirtschaftliche Entwicklung, Konjunkturprognosen wurden deutlich nach unten revidiert. Darüber hinaus stellt ein mögliches komplettes Ausbleiben aller Lieferungen von Energieträgern aus Russland ein erhebliches Risiko dar. Über die zu erwartenden Folgen ist viel diskutiert worden. Wenn es um die Auswirkungen eines Lieferstopps von Gas geht, kommen bisherige Berechnungen auf Basis ökonomischer Modelle zu uneindeutigen Ergebnissen mit einer Spannbreite von einem verkraftbaren Schock (Bachmann et al., 2022) bis hin zu massiven und dauerhaften wirtschaftlichen Schäden (Krebs, 2022).

## Abstract (en)

Based on data from a representative German establishment survey, we assess the ramifications of potentially cutting off the gas supply from Russia. Moreover, we analyse how rising energy costs and supply chain disruptions are so far related to the probability of firms adjusting prices, output and employment. A large share of businesses, especially energy-intensive ones, expect a partial or full reduction in output levels should they no longer be supplied with gas. Fourteen percent of businesses report that their production already decreased due to the Russia-Ukraine war and even more expect such a decrease in the future. Similarly, many businesses report price increases. Output and price adjustments are more likely to occur among businesses reporting rising energy costs or supply chain bottlenecks. Some have taken personnel measures, but overall, employment has remained robust so far.

Wir untersuchen die Auswirkungen eines möglichen Lieferstopps von russischem Gas auf Basis einer repräsentativen Betriebsbefragung. Diese liefert Informationen dazu, wie stark die Betriebe die Auswirkungen auf die Produktion einschätzen und ob aus deren Sicht das ausbleibende Gas durch andere Energieträger ersetzt werden könnte. Neben einem Gas-Lieferstopp untersuchen wir die Steigerung der Energiepreise sowie Probleme mit Lieferketten und deren Auswirkungen auf Preise, Produktion und Personal der Betriebe. Unsere Datengrundlage ist die Befragung „Betriebe in der Covid-19-Krise“ des Instituts für Arbeitsmarkt- und Berufsforschung (IAB), in der seit August 2020 regelmäßig rund 2.000 Betriebe zur aktuellen Situation befragt werden (Backhaus et al., 2021; Bellmann et al., im Erscheinen). Im Zuge der russischen Invasion der Ukraine wurden außerdem Fragen zu den wirtschaftlichen Auswirkungen des Kriegs aufgenommen. Die hier dargestellten gewichteten Ergebnisse basieren auf der 23. Welle der Befragung, an der zwischen dem 2. und 20. Mai 2022 knapp 1.980 Betriebe teilgenommen haben. Die Stichprobe entstammt der Betriebsdatei der Bundesagentur für Arbeit und ist repräsentativ für alle privatwirtschaftlichen Betriebe mit mindestens einem sozialversicherungspflichtig Beschäftigten.

**Christian Kagerl** ist wissenschaftlicher Mitarbeiter der Forschungsgruppe des Direktors am Institut für Arbeitsmarkt- und Berufsforschung (IAB) in Nürnberg.

**Dr. Michael Moritz**, **Dr. Jens Stegmaier** und **Dr. Ignat Stepanok** (Leiter der Arbeitsgruppe Folgen der Coronapandemie) sind wissenschaftliche Mitarbeiter am IAB.

**Dr. Duncan Roth** ist Leiter der Nachwuchsforschungsgruppe Berufe und Erwerbsverläufe am IAB.

**Prof. Dr. Enzo Weber** ist Forschungsbereichsleiter am IAB und lehrt an der Universität Regensburg.

## Folgen eines möglichen Lieferstopps für Gas

Abbildung 1 zeigt das Ausmaß der Verwendung von verschiedenen Energieträgern, die die Betriebe außer Strom einsetzen, je nachdem, ob sich Betriebe selbst als energieintensiv einschätzen oder nicht. Es kann um den Einsatz von Energie für Maschinen gehen, das Heizen von Gebäuden, die Nutzung von Kraftstoffen für Fahrzeuge oder als Vorprodukt (z. B. in der chemischen Industrie). Insgesamt verwenden fast drei Viertel der Betriebe Kraftstoffe wie Diesel, gefolgt von Erdgas, das von knapp der Hälfte der Betriebe (48 %) verbraucht wird. Während Erd- oder Heizöl in mehr als jedem fünften Betrieb verwendet wird, kommt Stein- oder Braunkohle nur in sehr wenigen Fällen zum Einsatz. 14 % der Betriebe nutzen auch andere Energieträger, die unter der Kategorie „Andere“ zusammengefasst werden, beispielsweise Biomasse. Die Nutzungsintensität der einzelnen eingesetzten Energieträger wurde nicht erfragt.

Abbildung 1

**Einsatz von Energieträgern außer Strom**

in %, Anteil aller Betriebe

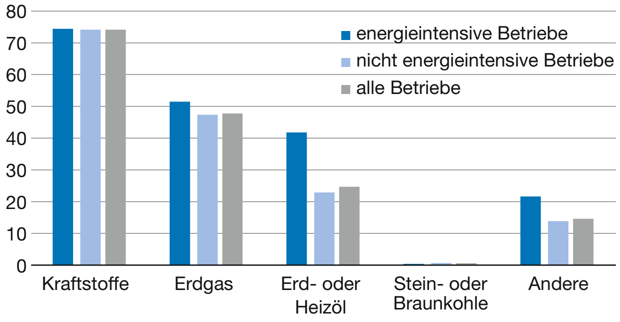

Quelle: IAB-Betriebsbefragung „Betriebe in der Covid-19-Krise“ (Welle 23); N=1.860 Betriebe; Mehrfachnennungen möglich; eigene Berechnungen.

Abbildung 2

**Folgen eines Erdgaslieferstopps auf die Produktion für energieintensive und nicht-energieintensive Betriebe**

Anteile in % der Betriebe, die Erdgas nutzen

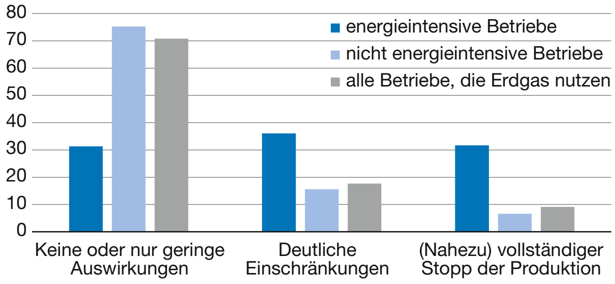

Quelle: IAB-Betriebsbefragung „Betriebe in der Covid-19-Krise“ (Welle 23); N=963 Betriebe; eigene Berechnungen.

Gemäß dem Erneuerbare-Energien-Gesetz (EEG) können Betriebe in energieintensive und nicht energieintensive unterteilt werden. Energieintensive Betriebe sind von der EEG-Umlage teilweise befreit. Berechtigt sind Unternehmen des produzierenden Gewerbes sowie Schienenbahnen, die jährlich mindestens 1 Gigawattstunde Strom verbrauchen und Stromkosten in Höhe von mindestens 14 % der Bruttowertschöpfung aufweisen. Nach Selbstauskunft bezeichnen sich 9 % aller Betriebe als energieintensiv, im Verarbeitenden Gewerbe ist der Anteil mit fast 17 % annähernd doppelt so hoch. Beim Einsatz von Energieträgern fällt auf, dass energieintensive Betriebe vor allem Erd- oder Heizöl anteilig mehr nutzen (vgl. Abbildung 1), während die anderen Energieträger zu ähnlichen Anteilen verwendet werden wie in nicht energieintensiven Betrieben. Wenn man die Kategorie „Andere“ als einen Energieträger zählt, dann berichten über 11 % aller Betriebe, dass sie drei oder mehr Energieträger nutzen, 49 %, dass sie zwei verbrauchen und 29 %, dass sie nur einen Energieträger neben Strom nutzen. Auffällig ist das Verarbeitende Gewerbe, wo die Zahl der Betriebe, die drei oder mehr Energieträger nutzen, bei rund 20 % liegt und damit deutlich höher als der Durchschnitt. Von zwei Energieträgern berichten rund 62 % der Betriebe im Verarbeitenden Gewerbe und 13 % von nur einem Energieträger.

Deutschland deckt bislang einen wesentlichen Teil des Bedarfs an Erdgas durch Importe aus Russland. Im Jahr 2021 betrug der Anteil der Gaslieferungen aus Russland 55 %, Ende April 2022 35 %. Die Bundesregierung plant, diesen Anteil weiter zu reduzieren. Trotzdem hätte ein möglicher Lieferstopp oder eine deutliche Reduzierung der Liefermengen derzeit spürbare Konsequenzen für die gesamte Wirtschaft, da viele Betriebe Erdgas nutzen, im Verarbeitenden Gewerbe sogar rund 60 %. Von den Betrieben, die Erdgas nutzen, berichten rund 70 %, dass ein Szenario, in dem sie nicht mehr mit Erdgas beliefert würden, keine oder nur geringe Auswirkungen auf die Produktion hätte (vgl. Abbildung 2). Eine deutliche Einschränkung der Produktion käme bei 18 % vor und knapp über 9 % berichten über einen vollständigen Stopp der Produktion. Im Verarbeitenden Gewerbe sind die Zahlen der negativen Auswirkungen im Vergleich zum Durchschnitt höher: Rund 19 % berichten von einer deutlichen Reduzierung und fast 15 % von einem kompletten Stopp der Produktion. Ebenso sind die negativen Auswirkungen auf die Produktion bei den energieintensiven Betrieben wesentlich höher: 36 % rechnen mit einer deutlichen Reduzierung und fast 32 % mit einem kompletten Stopp der Produktion. Die entsprechenden Anteile weisen bei den nicht energieintensiven Betrieben mit rund 16 % und 7 % signifikant niedrigere Werte auf.

Mit knapp 29 %, die deutliche Einschränkungen voraussehen, und fast 13 %, die von einem nahezu kompletten Produktionsausfall ausgehen, sind die Betriebe in Ostdeutschland zudem spürbar pessimistischer gestimmt. Weiterhin besteht auch ein Zusammenhang zwischen Betriebsgröße und den erwarteten Folgen eines Erdgas-Engpasses: Größere Betriebe prognostizieren im Durchschnitt gravierendere Auswirkungen auf ihre Produktion. Das im Zuge von Lieferreduktionen fehlende Erdgas könnte vollständig von nur 16 % der Betriebe durch andere Stoffe ersetzt werden. Zu einer teilweisen Substitution von Erdgas sehen sich rund 20 % der Betriebe imstande, die überwiegende Mehrheit von 58 % sieht kurzfristig keine Ersatzmöglichkeiten. Insbesondere energieintensive Betriebe und Betriebe in Ostdeutschland haben überproportional keine Option, Erdgas zu ersetzen.

## Maßnahmen wegen gestiegener Energiekosten

Jenseits der Frage, wie ein potenzieller Lieferstopp von Erdgas auswirken könnte, hat der Krieg in der Ukraine bereits jetzt zu wirtschaftlichen Schwierigkeiten für die Betriebe in Deutschland geführt. Diese Probleme dürften sich einerseits aus den steigenden Energiekosten ergeben – hiervon ist potenziell jeder Betrieb betroffen – und anderseits aus der Tatsache, dass die Lieferketten einiger Betriebe durch den Krieg gestört oder ganz unterbrochen wurden. Dabei ist zu bedenken, dass der Bezug von Vorleistungen oder Zwischenprodukten bereits vor Ausbruch des Kriegs durch die Coronapandemie mitunter stark beeinträchtigt war.

Um die wirtschaftliche Belastung der Betriebe durch den Krieg in der Ukraine einschätzen zu können, wurden die Betriebe gefragt, ob a) die Produktion bzw. die Erbringung von Dienstleistungen infolge des Kriegs reduziert wurde und ob b) die Betriebe ihre Preise erhöhen mussten. Dabei konnte angegeben werden, dass dies bereits geschehen ist oder dass ein Produktionsrückgang bzw. eine Preiserhöhung erwartet wird. Ein weiterer Fragenkomplex c) erfasste, ob Personalmaßnahmen[1] in Reaktion auf die wirtschaftlichen Folgen des Kriegs ergriffen wurden. In Tabelle 1 werden diese betrieblichen Reaktionen nach Wirtschaftszweigen gegliedert dargestellt. Bei allen Betrieben ist zu erkennen, dass knapp 12 % angeben, auf Personalmaßnahmen zurückzugreifen. Dies bezieht sich zum größten Teil auf reduzierte Arbeitszeiten und den Abbau von Überstunden. Daneben weisen die Befragten darauf hin, dass Einstellungen zurückgefahren wurden. Besonders häufig gaben Betriebe in den Bereichen Verkehr und Gastgewerbe (etwa 23 %) an, dass sie Personalmaßnahmen ergreifen mussten, auch im Verarbeitenden Gewerbe war dies bei immerhin 17 % der Fall. Bei den beiden zuerst genannten Branchen ist jedoch zu berücksichtigen, dass die zur Verfügung stehenden Fallzahlen dazu führen, dass die statistische Unsicherheit bei den berichteten Ergebnissen etwas höher ausfällt als in den anderen betrachteten Branchen.

Zu einem Rückgang der Produktion kam es bei 14 % aller Betriebe, etwa jeder fünfte Betrieb gibt zudem an, dass ein solcher Rückgang erwartet wird. Im Einzelnen ist für bereits erfolgte Reduktionen zu erkennen, dass wieder die Branchen Verkehr und Gastgewerbe relativ hohe Werte aufweisen, wohingegen dies im Bereich des Bildungs-, Gesundheits- und Sozialwesens praktisch kein Problem darstellt. Im Verarbeitenden Gewerbe sowie im Baugewerbe fällt auf, dass zwar nur etwa 14 % bzw. 18 % angeben, dass es bereits zu einem entsprechenden Rückgang kam, jedoch sagen weitere 33 % bzw. 39 %, dass ein solcher Produktionsrückgang zu erwarten ist. Von Preiserhöhungen, die von den Betrieben auf den Krieg zurückgeführt werden, wird am häufigsten berichtet: Fast jeder zweite Betrieb – 45 % – gibt an, dass dies bereits geschehen ist, weitere 27 % geben an, dass sie eine solche Erhöhung erwarten. Das Bauhauptgewerbe weist mit 77 % am häufigsten auf entsprechende Preiserhöhungen hin, überdurchschnittliche Werte finden sich ferner wieder in den Bereichen Verkehr und Gastgewerbe sowie im Verarbeitenden Gewerbe. Insgesamt ist nur im Dienstleistungsbereich sowie bei den Betrieben des Bildungs-, Gesundheits- und Sozialwesens zu erkennen, dass immerhin rund die Hälfte der Betriebe Preise infolge des Krieges weder erhöht hat noch davon ausgeht, diese erhöhen zu müssen. Die Betroffenheit von erhöhten Ausgaben für Energieträger seit Ausbruch des Krieges wurde mit einer kurzen Ja-oder-Nein-Frage erhoben. In einigen Branchen gaben die Befragten vermehrt an, dass sie keine Auskunft geben können. Daher weisen wir hier den Weiß-nicht-Anteil gesondert aus. Die Betroffenheit von Lieferengpässen infolge des Kriegs in der Ukraine werden mit einer längeren Frage ermittelt, wobei die Lieferengpässe z. B. in Gestalt von teilweisen oder vollständigen Ausfällen oder auch verspäteter Zulieferung der bestellten Vorleistungen oder Zwischenprodukte auftreten können.

In Tabelle 2 ist zu sehen, dass insgesamt die Mehrheit der Betriebe, rund 70 %, von erhöhten Energiekosten berichtet. Erhöhte Werte ergeben sich dabei insbesondere für tendenziell energieintensivere Branchen wie das Bauge-

Tabelle 1

**Maßnahmen der Betriebe in Reaktion auf die wirtschaftlichen Effekte des Ukrainekriegs**

Anteile in % der Betriebe nach Branche

| | Rückgang der Produktion | | | Preiserhöhungen | | | Personalmaßnahmen | |
|---|---|---|---|---|---|---|---|---|
| | Bereits erfolgt | Erwartet | Nein | Bereits erfolgt | Erwartet | Nein | Bereits erfolgt | Nein |
| Verarbeitendes Gewerbe | 14 | 33 | 53 | 67 | 19 | 14 | 17 | 83 |
| Baugewerbe | 18 | 39 | 43 | 77 | 21 | 2 | 14 | 86 |
| Groß- und Einzelhandel | 18 | 22 | 60 | 55 | 30 | 15 | 13 | 87 |
| Verkehr | 26 | 14 | 60 | 75 | 17 | 8 | 22 | 77 |
| Gastgewerbe | 23 | 25 | 52 | 73 | 21 | 6 | 23 | 77 |
| Dienstleistungen | 12 | 13 | 75 | 23 | 29 | 47 | 9 | 91 |
| Bildungs-, Gesundheits- und Sozialwesen | 1 | 11 | 87 | 13 | 32 | 55 | 5 | 95 |
| Gesamt | 14 | 21 | 66 | 45 | 27 | 28 | 12 | 88 |

Quelle: IAB-Betriebsbefragung „Betriebe in der Covid-19-Krise“ (Welle 23); N=1.849 Betriebe; eigene Berechnungen.

1 Die Betriebe hatten die Möglichkeit anzugeben, ob Beschäftigten gekündigt, Einstellungen verringert oder ausgesetzt, Kurzarbeit angemeldet, Leiharbeitskräfte oder Arbeitszeit bzw. Überstunden reduziert wurden. Die Rubrik „Personalmaßnahmen“ in der Tabelle gibt an, ob die Befragten mindestens eine dieser Maßnahmen genannt haben. Die wichtigsten Personalmaßnahmen werden im Text benannt, sind aber nicht im Einzelnen in der Tabelle aufgeführt.

Tabelle 2

**Betriebe, die angeben, gestiegenen Energiekosten oder Lieferengpässen ausgesetzt zu sein**

Anteile in % der Betriebe nach Branche

| | Gestiegene Energiekosten | | | Lieferengpässe infolge des Krieges | | | |
|---|---|---|---|---|---|---|---|
| | Ja | Nein | Weiß nicht | Ja | Nein | Unsicher | Keine Vorleistungen |
| Verarbeitendes Gewerbe | 78 | 20 | 3 | 51 | 28 | 9 | 13 |
| Baugewerbe | 87 | 11 | 2 | 34 | 25 | 12 | 29 |
| Groß- und Einzelhandel | 69 | 23 | 8 | 33 | 26 | 14 | 27 |
| Verkehr | 77 | 15 | 8 | 19 | 25 | 2 | 54 |
| Gastgewerbe | 74 | 22 | 4 | 41 | 18 | 3 | 38 |
| Dienstleistungen | 62 | 29 | 9 | 9 | 18 | 5 | 68 |
| Bildungs-, Gesundheits- und Sozialwesen | 61 | 26 | 14 | 13 | 24 | 4 | 60 |
| Gesamt | 70 | 23 | 7 | 25 | 22 | 8 | 45 |

Weiß nicht = Befragte/r kann keine Angabe machen. Unsicher: Betrieb hat Lieferengpässe, ist aber unsicher, ob diese Probleme auf den Krieg in der Ukraine zurückzuführen sind. Auf eine Darstellung der Kategorie „Weiß nicht" wird aufgrund der sehr geringen Inzidenz bei den Lieferengpässen verzichtet.

Quelle: IAB-Betriebsbefragung „Betriebe in der Covid-19-Krise" (Welle 23); N=1.914 Betriebe; eigene Berechnungen.

werbe (etwa 87 %), das Verarbeitende Gewerbe und im Bereich Verkehr (78 % und 77 %). Für den Dienstleistungsbereich ist dagegen, wie auch im Bereich des Bildungs-, Gesundheits- und Sozialwesens, ein unterdurchschnittlicher Wert zu beobachten. Bezüglich der Lieferengpässe kann der Tabelle entnommen werden, dass etwas mehr als die Hälfte aller Betriebe angibt, Vorleistungen oder Zwischenprodukte zu beziehen. Weitere knapp 25 % und damit fast die Hälfte aller Betriebe, die Vorleistungen beziehen, können Zulieferprobleme erkennen, die nach Einschätzung der Befragten auf den Krieg in der Ukraine zurückzuführen sind. Ca. 8 % der Betriebe berichten zudem von Schwierigkeiten in ihren Zulieferketten, bei denen sie sich unsicher sind, ob der Krieg die Ursache ist. Mit Abstand am häufigsten berichten von solchen Problemen die Betriebe des Verarbeitenden Gewerbes, wo mehr als die Hälfte aller Betriebe entsprechende Schwierigkeiten hat. Danach folgt – etwas überraschend – das Gastgewerbe. Eine Erklärung für den erhöhten Anteil in dieser Branche besteht aber in der zwischenzeitlichen Verknappung von Grundnahrungsmitteln wie Getreide oder Öl, die ebenfalls auf den Krieg in der Ukraine zurückzuführen ist.

Inwiefern ein Zusammenhang zwischen gestiegenen Energiekosten sowie Lieferengpässen auf der einen Seite und bereits vorgenommenen oder geplanten betrieblichen Maßnahmen auf der anderen besteht, ob also Betriebe, die von höheren Ausgaben für Energie oder von gestörten Lieferketten berichten, auch häufiger Anpassungen bei Personal, Produktion oder Preisen vorgenommen haben oder planen, untersuchen wir mit einer Regressionsanalyse. Für die Schätzung des Zusammenhangs nutzen wir im Fall der Produktions- und der Preisanpassungen jeweils ein multinomiales und im Fall der Personalanpassungen ein einfaches logistisches Modell. Die abhängigen Variablen sind Indikatoren dafür, ob ein Betrieb die Produktion infolge des Kriegs in der Ukraine reduziert bzw. Preise erhöht hat, dies plant oder keine solche Maßnahme ergriffen hat. Bei den Personalmaßnahmen unterscheiden wir zwischen Betrieben, die mindestens eine der oben genannten (vgl. Fußnote 1) Personalmaßnahmen vorgenommen haben, und solchen, die dies nicht getan haben. Bei den erklärenden Variablen differenzieren wir zunächst zwischen Betrieben mit und ohne höheren Energieausgaben seit Kriegsbeginn. Anhand der geschätzten Koeffizienten dieser Variable lassen sich Rückschlüsse darüber ziehen, ob es einen Zusammenhang zwischen höheren Energiekosten und den verschiedenen Anpassungen gibt. Da aber sowohl die Betroffenheit durch gestiegene Ausgaben für Energie als auch die Entscheidung über Anpassungen von Personal, Produktion und Preisen von anderen Faktoren beeinflusst werden können, berücksichtigen wir noch eine Reihe weiterer Kontrollvariablen. Zu diesen gehören betriebliche Strukturmerkmale wie der Wirtschaftszweig, die Betriebsgröße und der -standort (Ost- bzw. Westdeutschland). Weiterhin kontrollieren wir dafür, ob sich ein Betrieb als energieintensiv einschätzt und ob seine Geschäftstätigkeit vorwiegend innerhalb oder außerhalb Deutschlands liegt. Schließlich untersuchen wir auch den Zusammenhang mit Lieferengpässen. Hierfür nehmen wir Indikatoren auf, die angeben, ob es in einem Betrieb infolge des Kriegs zu Problemen beim Bezug von Vorleistungen oder Zwischenprodukten gekommen ist.

Tabelle 3 enthält die durchschnittlichen marginalen Effekte der erklärenden Variablen für jede Ergebnisvariable. Der Analyse zufolge besteht ein positiver Zusammenhang zwischen gestiegenen Energieausgaben und der Wahrscheinlichkeit für die verschiedenen Anpassungsmaßnahmen. Nach Berücksichtigung der anderen Kontrollvariablen weisen Betriebe mit höheren Energieausgaben im Vergleich zu den anderen eine um knapp 7 Prozentpunkte höhere Wahrscheinlichkeit auf, dass es bereits zu einem Rückgang der Produktion gekommen ist. Im Fall der Preiserhöhungen beträgt die entsprechende Differenz 12 Prozentpunkte und bei den Personalmaßnahmen 8 Prozentpunkte. Diese Unterschiede sind jeweils statistisch signifikant und konzentrieren sich in Betrieben, die nur einen Energieträger nutzen und daher über geringere Ausweichmöglichkeiten verfügen. Bei den geplanten Anpassungen zeigt sich sowohl bei der Produktions- als auch bei der Preisanpassung ein kleinerer positiver Zusammenhang, der jedoch statistisch nicht signifikant ist. Betriebe, die

Tabelle 3

**Anpassungen von Produktion, Preisen und Personal – durchschnittliche marginale Effekte**

| | Rückgang der Produktion | | | Preiserhöhung | | | Personalmaßnahmen |
|---|---|---|---|---|---|---|---|
| | Bereits erfolgt | Erwartet | Nein | Bereits erfolgt | Erwartet | Nein | Bereits erfolgt |
| Energieausgaben (Referenz: nicht gestiegen) | | | | | | | |
| Gestiegen | 0,068*** (0,025) | 0,044 (0,032) | 0,113*** (0,035) | 0,123*** (0,036) | 0,016 (0,037) | -0,139*** (0,033) | 0,075*** (0,020) |
| Keine Angabe/Weiß nicht | -0,001 (0,044) | 0,014 (0,061) | -0,013 (0,063) | 0,021 (0,067) | -0,040 (0,068) | 0,019 (0,053) | 0,040 (0,038) |
| Energieintensität (Referenz: nicht energieintensiv) | | | | | | | |
| Energieintensiv | -0,028 (0,032) | 0,155*** (0,052) | 0,127*** (0,053) | 0,090* (0,51) | -0,036 (0,050) | -0,054 (0,053) | 0,052 (0,038) |
| Keine Angabe/Weiß nicht | 0,032 (0,063) | 0,169** (0,080) | -0,201** (0,082) | -0,001 (0,077) | 0,064 (0,087) | -0,063 (0,053) | 0,016 (0,052) |
| Lieferengpässe infolge des Kriegs in der Ukraine (Referenz: keine Probleme beim Bezug von Vorleistungen) | | | | | | | |
| Probleme beim Bezug von Vorleistungen | 0,179*** (0,035) | 0,176*** (0,039) | -0,355*** (0,044) | 0,281*** (0,046) | -0,086* (0,049) | -0,196*** (0,037) | 0,118*** (0,032) |
| Unsicher ob Probleme auf Krieg zurückzuführen sind | 0,044 (0,046) | 0,074 (0,048) | -0,118* (0,060) | 0,025 (0,058) | 0,054 (0,072) | -0,079 (0,061) | 0,060 (0,044) |
| Kein Bezug von Vorleistungen | 0,037 (0,027) | 0,078** (0,033) | -0,115*** (0,039) | 0,093** (0,039) | 0,166*** (0,041) | 0,073* (0,037) | 0,014 (0,024) |
| Beobachtungen | 1.783 | 1.783 | 1.783 | 1.789 | 1.789 | 1.789 | 1.896 |

Die Tabelle enthält die durchschnittlichen marginalen Effekte und deren robuste Standardfehler in Klammern. ***/**/* kennzeichnen statistische Signifikanz am 1 %-/5 %-/10 %-Niveau. Neben den oben aufgeführten Variablen wird in der Schätzung für den Wirtschaftszweig, die Betriebsgröße, den Standort (Ost- bzw. Westdeutschland) sowie für den Exportstatus des Betriebs kontrolliert.

Quelle: IAB-Betriebsbefragung „Betriebe in der Covid-19-Krise“ (Welle 23); eigene Berechnungen.

keine Angaben zu den Energieausgaben machen können, unterscheiden sich mit Blick auf die genannten Anpassungen nicht von denen ohne gestiegene Energiekosten.

Energieintensive Betriebe haben ceteris paribus zwar nicht häufiger ihre Produktion reduziert als nicht energieintensive Betriebe, weisen dagegen aber eine um knapp 16 Prozentpunkte höhere Wahrscheinlichkeit auf, die Produktion in Zukunft zurückfahren zu wollen. Ein ähnlich starker Zusammenhang ergibt sich für Betriebe, die keine Angabe zu ihrer Energieintensität machen können. Ein möglicher Grund ist, dass es sich bei diesen Betrieben im Durchschnitt um solche mit einem hohen Energieverbrauch handelt. Bei den Preis- und Personalmaßnahmen zeigt sich zwar auch ein positiver Zusammenhang mit der Energieintensität der Betriebe, dieser ist jedoch nur schwach bzw. statistisch nicht signifikant. Somit findet sich kein Nachweis dafür, dass energieintensive Betriebe – nach Berücksichtigung von höheren Energiekosten und Lieferengpässen – mit Blick auf Personal und Preise anders reagiert haben als nicht energieintensive Betriebe. Für die Frage, ob Betriebe Anpassungen bei Produktion, Preisen oder Personal vorgenommen haben, sind jedoch nicht nur höhere Energiepreise von Bedeutung. Den Ergebnissen zufolge besteht für jede Größe außerdem ein statistisch signifikanter Zusammenhang mit der Existenz von Lieferproblemen. So weisen Betriebe, die von Problemen bei der Zulieferung von Vorleistungen oder Zwischenprodukten berichten, gegenüber Betrieben ohne solche Probleme eine um fast 30 Prozentpunkte erhöhte Wahrscheinlichkeit auf, Preise erhöht zu haben. Im Fall der Produktionsreduktion liegt dieser Unterschied bei knapp 18 Prozentpunkten und bei den Personalmaßnahmen bei knapp 12 Prozentpunkten. Betriebe mit Problemen beim Bezug von Vorleistungen geben darüber hinaus auch häufiger an, eine Drosselung ihrer Produktion zu planen. Weniger eindeutig ist das Bild bei Betrieben, die keine Vorleistungen oder Zwischenprodukte beziehen. Im Vergleich zu Betrieben, die solche Leistungen beziehen, aber keine Lieferprobleme haben, gibt diese Gruppe an Betrieben häufiger an, Produktionsanpassungen zu planen und Preiserhöhungen vorgenommen zu haben.

Aus Tabelle 1 geht hervor, dass manche Betriebe ihre Produktion infolge des Kriegs in der Ukraine zwar noch nicht reduziert haben, dies aber für die Zukunft planen. Eine geringere Produktion kann wiederum den Bedarf nach Arbeit in einem Betrieb reduzieren und daher potenziell Folgen für die Beschäftigung haben. In einer weiteren Auswertung (ohne Tabelle) untersuchen wir daher, ob Betriebe, die ihre Produktion reduziert haben, bereits Anpassungen bei ihrem Personal vorgenommen haben. Dafür verwenden wir dieselben Kontrollvariablen wie in Tabelle 3. Ceteris paribus zeigt sich, dass Betriebe, die ihre Produktion bereits reduziert haben, eine um etwa 30 Prozentpunkte höhere Wahrscheinlichkeit aufweisen, auch mindestens eine Personalmaßnahme ergriffen zu haben, als Betriebe ohne Produktionsanpassung. In Betrieben, die eine Drosselung der Produktion planen, gibt es eine um knapp 9 Prozentpunkte höhere Wahrscheinlichkeit für Personalmaßnahmen – ein Teil dieser Betriebe hat somit womöglich schon personelle Anpassungen vorweggenommen. Sollte es dazu kommen, dass Betriebe ihre für die Zukunft geplante Senkung der Produktion umsetzen, ist damit zu rechnen, dass sich daraus auch weitere Anpassungen beim Personal ergeben. In zusätzlichen Regressionen wurde auch die Betroffenheit der Betriebe durch die Coronakrise aufgenommen. Dabei zeigt sich, dass Betriebe infolge des Kriegs signifikant häufiger Personalmaßnahmen ergreifen, wenn sie bereits negativ durch Corona betroffen waren. Bei Kumulation von Kriseneffekten ergeben sich also stärkere Auswirkungen des Kriegs.

## Fazit

Unsere Auswertung der betrieblichen Informationen zur Energiekrise ergab, dass die Risiken eines Gas-Lieferstopps für die deutsche Wirtschaft hoch sind: Die meisten Betriebe, die Erdgas nutzen, können es kurzfristig nicht ersetzen. Viele erwarten einen deutlichen Produktionsrückgang, falls sie gar nicht mehr beliefert werden. Hier ist zu berücksichtigen, dass die meisten Betriebe auch im Fall eines russischen Lieferstopps weiter versorgt würden, aber insgesamt käme man um eine Rationierung in bestimmtem Umfang nicht herum. Dabei ist die Energiekrise auch ohne Lieferstopp schon in vollem Gange. Unsere Ergebnisse zeigen bereits jetzt deutliche Wirkungen der Energiepreissteigerungen auf die Produktion und die Preise. Auch Hutter und Weber (2022) schätzen, dass die Produktion im Verarbeitenden Gewerbe schon im März infolge der Energiekrise um rund 1 % sank. Dabei stellt die empirische Forschung zu Energiepreisschocks (z. B. Kilian, 2008) die vollen ökonomischen Wirkungen erst nach einigen Quartalen fest. Dazu passt unser Befund, wonach gerade die energieintensiven Betriebe einen Rückgang ihrer Produktion noch erwarten. Zudem könnten die Energiekosten noch weiter steigen, weil etwa Lieferverträge erst nach und nach angepasst werden. Falls im Notfallplan Gas die Alarmstufe ausgerufen wird, könnten Versorger die gestiegenen Kosten sogar kurzfristig auf die Kundschaft abwälzen. Das macht deutlich, dass die Energiekrise die wirtschaftliche Entwicklung länger beeinträchtigen dürfte. Produktionsrückgänge sind besonders kritisch, wenn sie weitere Folgewirkungen in der Wirtschaft auslösen. Energieintensive Betriebe gehören meist zur Grundstoffindustrie und stehen damit am Anfang von Lieferketten. Auch in unseren Ergebnissen wird deutlich, dass Probleme mit den Lieferketten zu Produktionsrückgängen und Preiserhöhungen führen. Üblicherweise führt das zu weiterer Kurzarbeit (Hummel et al., 2022). Wirtschaftspolitische Instrumente sollten deshalb die Fortführung der Produktion kurzfristig unterstützen, dabei aber die Anreize der hohen Preise für Energieeffizienz erhalten. Ein Instrument dafür sind Produktionsprämien (Weber, 2022a,b), wie sie auch als Kostenzuschüsse für energieintensive Betriebe von der Bundesregierung beschlossen wurden.
